# Occupational and Environmental Exposures Associated with Testicular Germ Cell Tumours: Systematic Review of Prenatal and Life-Long Exposures

**DOI:** 10.1371/journal.pone.0077130

**Published:** 2013-10-14

**Authors:** Rémi Béranger, Charlotte Le Cornet, Joachim Schüz, Béatrice Fervers

**Affiliations:** 1 Unité Cancer et Environnement, Centre Léon Bérard, Lyon, France; 2 Section of Environment and Radiation, International Agency for Research on Cancer, Lyon, France; 3 Université Claude, Bernard, Lyon, France; University Hospital of Münster, Germany

## Abstract

**Background:**

Testicular germ cell tumours (TGCT) are the most common cancers in men aged between 15 and 44 years and the incidence has increased steeply over the past 30 years. The rapid increase in the incidence, the spatial variation and the evolution of incidence in migrants suggest that environmental risk factors play a role in TGCT aetiology. The purpose of our review is to summarise the current state of knowledge on occupational and environmental factors thought to be associated with TGCT.

**Methods:**

A systematic literature search of PubMed. All selected articles were quality appraised by two independent researchers using the ‘Newcastle-Ottawa Quality Assessment Scale’.

**Results:**

After exclusion of duplicate reports, 72 relevant articles were selected; 65 assessed exposure in adulthood, 7 assessed parental exposures and 2 assessed both. Associations with occupation was reported for agricultural workers, construction workers, firemen, policemen, military personnel, as well as workers in paper, plastic or metal industries. Electromagnetic fields, PCBs and pesticides were also suggested. However, results were inconsistent and studies showing positive associations tended to had lower quality ranking using the assessment scale (p=0.02).

**Discussion:**

Current evidence does not allow concluding on existence of any clear association between TGCT and adulthood occupational or environmental exposure. The limitations of the studies may partly explain the inconsistencies observed. The lack of association with adulthood exposure is in line with current hypotheses supporting the prenatal origin of TGCT. Future research should focus on prenatal or early life exposure, as well as combined effect of prenatal and later life exposure. National and international collaborative studies should allow for more adequately powered epidemiological studies. More sophisticated methods for assessing exposure as well as evaluating gene–environment interactions will be necessary to establish clear conclusion.

## Introduction

Testicular cancer is the most common cancer in men aged 15 - 44 years. Incidence rates have increased steeply in developed countries, with the highest incidence rates in Europe and in the USA [1]. Testicular germ cell tumours (TGCT) represent more than 90% of testicular cancer. Two main histological forms occur among young men: non-seminomas, which have an incidence that peaks at around 25 years old and seminomas that peak later, at around 35 years old. TGCTs in young adults should be distinguished from other rarer TGCTs histologies, which have different pathogenesis [2–4]: yolk sac tumours and immature teratomas occurring during childhood, and spermatocytic seminoma affecting mostly men over 50 years of age. The rapid increase and spatial disparities [1] of TGCT incidence as well as changes in incidence between first and second-generation immigrants [5–7] support a multifactorial origin of TGCT and in particular a role of environmental factors.

The possibility of an early life induction of TGCT is supported by the young age of cases, by the association with congenital abnormalities of the testis (cryptorchidism and hypospadias) and results from numerous experimental studies suggesting that seminomas and non-seminomas could have a common precursor – the carcinoma in situ cell [4,8]. Testicular Dysgenesis Syndrome (TDS) has been proposed as the common origin for TGCT (except spermatocytic seminomas), cryptorchidism, hypospadias, and several types of decreased sperm quality. This syndrome may be caused by abnormal development of Sertoli and Leydig cells in the foetal testis resulting in delayed differentiation of germ cells and lower testosterone serum level during *in-utero* life [9]. Rare mutations, e.g. SRY mutations, can cause Testicular Dysgenesis Syndrome (TDS) but in most cases no mutations have been identified. It has been suggested that perinatal exposure to endocrine disruptors with estrogenic and anti-androgenic properties may play a role, particularly in individuals with genetic susceptibility to Testicular Dysgenesis Syndrome (TDS) [10]. Although this concept of TDS is currently controversial, the hypothesis of a prenatal (or early life) origin of TGCT is widely accepted [11,12].

So far, no animal models expressing TGCT type of the young adult have been found, although cases of spermatocytic seminomas have been reported. Our knowledge about TGCT risk factors is therefore based on epidemiological research [2]. Among the potential environmental factors, pesticides, which were first suggested to be a possible risk factor for TGCT in 1984 [13], appear to be one of the most studied. Available literature reviews [3,14–16] focused mainly on adulthood exposures and missed several articles. These generally provided few details on study methodologies and limits. Our systematic review aimed to critically analyse and evaluate available evidence from epidemiological studies to examine prenatal as well as life-long environmental and occupational exposures associated to TGCT.

## Methods

### Literature search

We followed the PRISMA statement for systematic reviews and meta-analysis for literature search, study selection, data extraction and synthesis (Checklist S1). A systematic review protocol was formalized with epidemiologist advisors (protocol not registered). Two independent investigators (RB and CLC) searched PubMed to identify relevant epidemiological studies on occupational and environmental risk factors for TGCT published between 1^st^ January 1990 and 31^st^ December 2012. Individual lifestyles factors (e.g. drugs, physical activity, tobacco, marijuana) were not included in this review. 

The following search algorithm was used: (*"Testosterone/antagonists and inhibitors"[Mesh*]* OR "Endocrine Disruptors"[Mesh*]* OR "Pesticides"[Mesh*]* OR "Endocrine Disruptors"[TIAB*]* OR "Pesticides"[TIAB*]* OR "maternal exposure"[mesh*]* OR "environmental exposure"[mesh*]* OR "occupational diseases"[mesh*]* OR "occupations"[mesh*]) *AND* (*"Germinoma"[Mesh*]* OR "Testicular Neoplasms"[Mesh*]* OR "seminoma"[TIAB*]* OR "testicular dysgenesis syndrome"[tiab*]* OR "testicular cancer"[tiab*])*.*

Possibly relevant articles were selected through assessment of titles and abstract. Only original articles focusing on humans and written in English or French were kept in the review. Given the high survival rate of TGCT (more than 95% for localised tumours, 80% if metastatic), mortality studies lead to a selection of the population and were considered inappropriate for the purpose of our review [17]. To complete our literature search, we screened the reference lists of selected articles and related reviews. 

For each publication, we abstracted the following information: first author’s name; year of publication; journal; country of the studied population; study design; population size and characteristics (source, age structure, follow up, composition); approaches for exposure and outcome assessment; variable for stratification, groups matching or adjustment; and main results. When two or more publications reported data from the same study populations, we kept only the most detailed and/or the most recent publication. Publications with partially overlapping populations were retained when they provided complementary information.

### Quality appraisal

Two researchers (RB and CLC) independently assessed the methodological quality of each study using the “Newcastle-Ottawa Quality Assessment Scale” (NOS). (http://www.ohri.ca/programs/clinical_epidemiology/oxford.asp). This scale has nine items in three parts: selection (four items), comparability (two items) and outcome (for cohort design, three items) or exposure (for case-control design, three items). The highest quality score a paper can obtain is ‘9’. In the event of disagreement, BF and JS provided input to obtain a consensus. Different publications issued from a same study might have diverging Newcastle-Ottawa Quality Assessment Scale (NOS) scores if the methodological aspects changed (e.g. adjustment factors, method for exposure assessment).

### Statistical analyses

Statistical analyses were performed using SAS software package (version 9.3; SAS Institute Inc., Cary, NC, USA). The Newcastle-Ottawa Quality Assessment Scale (NOS) quality scores were compared using the Wilcoxon test.

## Results

We identified 265 articles published between 1^st^ January 1990 and 31^st^ December 2012. We excluded 189 reviews, editorials and animal studies, 2 articles published in a language other than English or French, 23 publications that were out of scope, and 6 mortality studies. By analysing the reference lists of the 45 remaining articles and existing reviews on TGCT [3,14–16], we identified 35 additional publications responding to our inclusion criteria. Overall, 80 studies were selected and checked for potential overlap.

**Figure 1:**
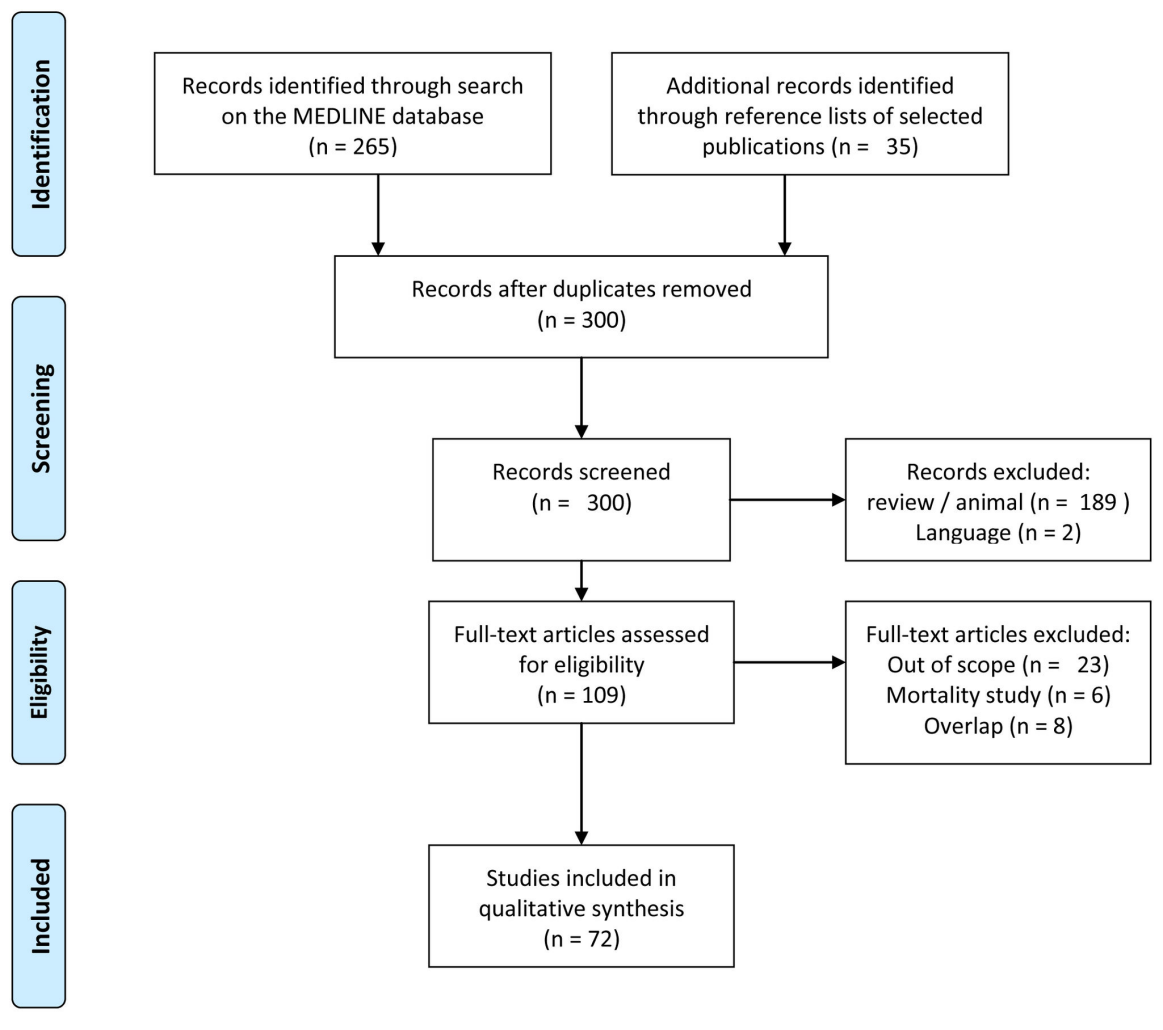
Flow diagram of identified articles published between 1990 and 2012.

14 of the 80 selected publications reported data from the same study populations [20-33]. We kept the 6 most detailed or most recent of these for analysis [18–23]. Publications with partially overlapping populations were retained because they provided complementary information [24–26]: [27–29]; [30–32]; [33,34];; [35–38]. In total, we analyzed data from 72 publications for this review. Figure **1** represents the flow diagram of the articles selection. 

The characteristics of the 72 selected articles and their NOS quality scores are summarized in Table **1** (two were ecological studies and thus not scored) [39,40]. The mean overall score was 6.3 out of 9 (2.6/4 for the selection part, 1.3/2 for comparability, 2.2/3 for outcome) with a high quality score (8 or 9) for eleven publications, an intermediate score (6 or 7) for 41 articles and a score of 5 or less for 18 publications. Studies published after 2000 were of higher quality than those published before 2000 (p< 0.01).

**Table 1:** Description of selected studies.

**Reference**	**Study design**	**Study population**	**Age limits**	**Exposure assessment**	**Diagnostic periods**	**Country**	**Quality assessment^a^**			
							**Sel. (4pt)**	**Com. (2pt)**	**Out. (3pt)**	**Total (9pt)**
Alavanja 2005 [72]	Cohort^b^	57311 exposed / 27 cases	No details^c^	Questionnaire	1993 - 2002	USA	3	2	2	**7**
Andersson 2003 [54]	Cohort^b^,^e^	65637 exposed / 49 cases	31 - 84	Registry	1971 - 1990	Sweden	3	2	3	**8**
Andersson 2012 [57]	Cohort^b^	18113 workers / 26 cases	No details	Registry	1958 - 2001	Sweden	2	1	3	**6**
Band 2001 [55]	Cohort^b^	28278 exposed / 23 cases	No details	Registry	1950 - 1992	Canada	3	1	3	**7**
Bates 2001 [18]	Cohort^b^	3668 exposed / 11 cases	No details	Registry	1977 - 1995	New Zealand	3	1	2	**6**
Dement 2003 [59]	Cohort^b^	13354 exposed / 19 cases	No details	Registry	1979 - 2000	New Jersey	3	1	3	**7**
Dich 1996 [73]	Cohort^b^	20025 exposed / 18 cases	No details	Registry	1965 - 1991	Sweden	3	1	3	**7**
Finkelstein 1998 [65]	Cohort^b^	20601 exposed / 23 cases	No details	Registry	1964 - 1995	Ontario	1	1	2	**4**
Fleming 1999 [74]	Cohort^b^	30155 exposed / 23 cases	18 - 89	Registry	1981 - 1993	Florida	3	1	3	**7**
Floderus 1999 [30]	Cohort^b^	1596959 men / 607 cases	20 - 70	Registry	1971 - 1984	Sweden	2	0	3	**5**
Frost 2011 [75]	Cohort^b^	62960 exposed / 102 cases	No details	Registry	1987 - 2004	UK	2	1	2	**5**
Giles 1993 [62]	Cohort^b^	2865 exposed / 2 cases	15 / +^d^	Registry	1980 - 1989	Melbourne	3	1	3	**7**
Grayson 1996 [67]	Cohort^b^	227203 exposed / 59 cases	No details	Registry	1975 - 1989	USA	2	1	2	**5**
Guo 2004 [33]	Cohort^b^,^e^	667121 workers / 387 cases	25 / +^d^	Registry	1971 - 1995	Finland	3	2	3	**8**
Guo 2005 [34]	Cohort^b^,^e^	667121 workers / 387 cases	25 / +^d^	Registry	1971 - 1995	Finland	3	2	3	**8**
Gustavsson 2004 [97]	Cohort^b^	8750 exposed / 8 cases	No details	Registry	1989 - 1999	Sweden	3	1	2	**6**
Hansen 1996 [43]	Cohort^b^	10059 exposed / 4 cases	No details	Questionnaire	1968 - 1986	Denmark	1	1	3	**5**
Helmfrid 2012 [81]	Cohort^b^	641 cancers / 7 testis cancer	No details	Registry	1960 - 2003	Sweden	3	1	3	**7**
Hobbesland 1999 [53]	Cohort^b^	5918 exposed / 13 cases	No details	Registry	1953 - 1991	Norway	1	1	3	**5**
Kelleher 1998 [76]	Cohort^b^	About 150000 men / 47 cases	16 - 65	Registry	1980 - 1990	Ireland	3	1	1	**5**
Kristensen 1996 [21]	Cohort^b^	166291 exposed / 158 cases	0 - 39	Registry	1965 - 1991	Norway	3	2	2	**7**
Kristensen 2000 [98]	Cohort^b^	47285 men and 36787 son exposed / 70 and 63 cases	0 - 70	Registry	1967 - 1995	Norway	2	2	2	**6**
Langard 2000 [47]	Cohort^b^	428 exposed / 1 cases	No details	Registry	1953 - 1993	Norway	3	1	3	**7**
Ma 2006 [63]	Cohort^b^	34796 exposed / 54 cases	18 / +^d^	Registry	1981 - 1999	Florida	2	1	3	**6**
Milanov 1999 [68]	Cohort	52963 person-year / 6 cases	No details	Registry	1964 - 1994	Bulgaria	1	1	3	**5**
Rix 1998 [56]	Cohort^b^	11130 men / 29 cases	No details	Registry	1943 - 1996	Denmark	3	1	3	**7**
Rodval 2003 [85]	Cohort^b^	About 14000 exposed / 2 cases	0 - 36	Registry	1958 - 1994	Sweden	3	1	2	**6**
Pollan 2001 [31]	Cohort^b^	1779646 men / 1189 cases	24 - 79	Registry	1970 - 1989	Sweden	3	2	3	**8**
Sigurdson 2003 [83]	Cohort^b^,^f^	20781 exposed / 16 cases	No details	Questionnaire	1983 - 1998	USA	2	2	3	**7**
Sonneveld 1999 [23]	Cohort^b^	7473676 men / 2591 cases	No limits	Registry	1989 - 1995	Netherland	3	1	2	**6**
Sulem 2003 [99]	Cohort^b^	3874 exposed / 0 cases	18 / +^d^	Registry	1968 - 1998	Iceland	2	1	3	**6**
Tynes 1992 [60]	Cohort^b^	37945 exposed / 41 cases	20 / +^d^	Registry	1961 - 1985	Norway	3	1	3	**7**
Yamane 2006 [71]	Cohort^b^,^f^	From 281604 to 489590 exposed / 354 cases	18 - 51	Registry	1989 - 2002	USA	2	2	3	**7**
Zandjani 1994 [77]	Cohort^b^	1756 exposed / 7 cases	No details	Registry	1953 - 1992	Norway	3	1	3	**7**
Bates 2007 [61]	Case-control^b,e,f^	70 cases / 804107 controls	21 - 80	Registry	1988 - 2003	California	2	2	2	**6**
Baumgardt-Elms 2002 [24]	Case-control	269 cases / 797 controls	15 - 69	Interview	1995 - 1997	Germany	3	2	2	**7**
Baumgardt-Elms 2005 [25]	Case-control	145 cases / 196 controls	15 - 69	GIS	1995 - 1997	Germany	3	1	3	**7**
Biggs 2008 [78]	Case-control	272 cases / 726 controls	18 - 44	Biol. sample	1999 - 2008	Washington	4	1	2	**7**
Bullman 1994 [41]	Case-control^b^	97 cases / 311 controls	28 / + ^d^	Registry	1982 - 1991	USA	1	0	2	**3**
Chia 2010 [27]	Case-control^f^	577 cases / 707 controls	18 - 45	Biol. sample	1988 - 2003	USA	4	2	3	**9**
Foley 1995 [66]	Case-control^b^	148 cases	17 - 49	Registry	1984 - 1989	UK	3	1	2	**6**
Giannandrea 2011 [42]	Case-control^e^	50 cases / 48 controls	18 - 45	Interview + biol. sample	2006 - 2008	Italia	2	2	2	**6**
Hansen 1999 [46]	Case-control^b^	3745 cases / 7490 controls	16 - 75	Registry	1970 - 1989	Denmark	3	2	2	**7**
Hardell 2006 [19]	Case-control	61 cases / 58 controls	18 - 45	Biol. Sample	1997 - 2000	Sweden	3	1	2	**6**
Hardell 2004 [49]	Case-control	791 paired cases and controls	20 - 75	Questionnaire	1993 - 1997	Sweden	3	1	2	**6**
Hayes 1990 [35]	Case-control	266 cases / 271 controls	18 - 42	Interview	1976 - 1981	Washington	2	2	2	**6**
Kardaun 1991 [36]	Case-control	308 cases / 288 controls, 225 and 212 mothers, respectively	18 - 42	Interview	1976 - 1981	Washington	2	1	2	**5**
Knoke 1998 [20]	Case-control^b^,^f^	134 cases / 371 controls	17 - 65	Registry	1990 - 1996	USA	2	2	1	**5**
Knight 1996 [51]	Case-control^e^	495 cases / 974 controls	16 - 59	Questionnaire	1987 - 1989	Ontario	3	2	1	**6**
Knight 1997 [87]	Case-control^e^	495 cases / 974 controls, 343 and 524 mothers, respectively	16 - 59	Questionnaire	1987 - 1989	Ontario	3	1	1	**5**
Marshall 1990 [82]	Case-control	18 cases / 259 controls	20 - 54	Registry	1974 - 1986	New York	2	1	1	**4**
McGlynn 2009 [29]	Case-control^f^	736 cases / 913 controls	18 - 45	Biol. Sample	1988 - 2003	USA	4	2	3	**9**
McGlynn 2008 [28]	Case-control^f^	739 cases / 915 controls	18 - 45	Biol. Sample	1988 - 2003	USA	4	2	3	**9**
Moller 1997 [80]	Case-control^f^,^g^	296 cases / 287 controls	16 - 42	Questionnaire	1986 - 1988	Denmark	3	2	1	**6**
Nori 2006 [44]	Case-control^e,f,g^	103 cases / 215 controls ; 63 and 123 mothers, respectively	18 / +^d^	Interview	1996 - 2003	Italia	2	2	1	**5**
Ohlson 2000 [22]	Case-control	148 cases / 314 controls	30 - 75	Questionnaire	1989 - 1992	Sweden	3	1	2	**6**
Rhomberg 1995 [52]	Case-control	165 cases / 187 controls	18 / +^d^	Interview	1971 - 1978	Germany	3	0	0	**3**
Ryder 1997 [69]	Case-control^b^	110 cases / 440 controls	15 - 59	Registry	1976 - 1994	UK	4	1	3	**8**
Stang 2003 [26]	Case-control^g^	269 cases / 797 controls	15 - 69	Interview	1995 - 1997	Germany	3	2	1	**6**
Stenlund 1997 [32]	Case-control^b^	134 cases / 1121 controls	25 -70	Registry	1985 - 1987	Sweden	3	2	2	**7**
Swerdlow 1991 [45]	Case-control	259 cases / 489 controls	10 / +^d^	Interview	1977 - 1981	UK	1	1	1	**3**
Tarone 1991 [37]	Case-control^g^	156 cases / 130 controls	18 - 42	Interview	1976 - 1981	Washington	2	2	2	**6**
Van der Eeden 1991 [38]	Case-control^f^,^g^	390 cases / 729 controls	20 - 69	Interview	1977 - 1984	Washington	4	2	2	**8**
Walchaert 2007 [48]	Case-control^g^	229 cases / 800 controls	20 - 45	Questionnaire	2002 - 2005	France	2	1	1	**4**
Yamane 2003 [70]	Case-control^b^,^f^	74 cases / 296 controls	23 - 55	Registry	1989 - 1999	USA	2	2	3	**7**
Zhang 1995 [84]	Case-control^e^,^g^	250 cases and control	15 / +^d^	Interview	1977 - 1980	New York	3	2	1	**6**
Behrens 2012 [50]	Nested case-control	169 cases / 988 controls	No details	Interview	1989 - 2006	Germany	4	2	2	**8**
Cohn 2010 [86]	Nested case-control^f^	15 cases / 45 controls	17 - 37	Biol. sample	1957 - 2000	California	3	2	3	**8**
Purdue 2009 [79]	Nested case-control	49 cases / 51 controls	No details	Biol. Sample	1972 - 1999	Norway	3	1	3	**7**
Koifman 2002 [39]	Ecological study	No details	0 - 49	N/A	1999 - 2000	Brazil	N/A	N/A	N/A	
Mills 1998 [40]	Ecological study^f^	No details	No details	N/A	1988 - 1992	California	N/A	N/A	N/A	
Davis 1993 [100]	Cluster	6 case / 340 controls	27 - 47	Interview	1979 - 1991	Washington	1	0	3	**4**

Abbreviations: sel. = Selection; Com. = comparability; out. = outcome. Biol. Sample = biological sample. N/A = not applicable; GIS = Geographic Information System.

“Questionnaire” means self-administered questionnaire, in contrast to “Interview”. All studies were stratified or adjusted on age but four [35,41,52,100].

a *The quality score was determined by using the Newcastle-Ottawa quality assessment scale*;

b Registry-based studies.

c *Information not provided by authors*

d *No upper age limit.*

e Adjustment on socioeconomic status

f *Adjustment on ethnicity or study focusing only on Caucasian*

g Adjustment on (or exclusion of) cryptorchidism

Of the 72 publications included in this review, 65 investigated exposure of the index subject (Table **S1**) and nine investigated parental exposure (Table **2**) (two focused both). No quality differences were found between these two types of studies (p=0.36). TGCT was the primary interest in 41 publications, while 31 investigated a broad range of cancers. Among the 23 studies investigating seminomas and non-seminomas separately, no risk factor appeared to be specific for one or other histologies. Studies reporting positive associations had lower NOS scores than those reporting negative results (6.02 versus 6.70, p = 0.02), mainly due to studies in which a positive association with TGCT was found for items other than the primary objective [20,41–45].

**Table 2:** Parental occupational and environmental exposures related to testicular germ cell cancer in offspring.

***Exposure categories***	***Ref.***	***Exposure***	***Timeofcasesexposure***	***All testicular cancer***	***Seminoma***	**Non-seminoma**
**Occupational exposures**						
**Agricultural workers**						
Agriculture, forestry	[36]	Paternal	Childhood	OR = 0.4 [0.1-0.9]	-	-
Agriculture, forestry	[36]	Paternal	Prenatal	OR = 0.9 [0.4-1.8]	OR = 0.7 [0.1-2.7]	-
Employed in agriculture	[80]	Maternal	Prenatal	OR = 1.23 [0.56-2.69]	OR = 1.34 [0.50-3.57]	OR = 1.32 [0.51-3.40]
Employed in agriculture with animals	[80]	Paternal	Prenatal	OR = 0.64 [0.42-0.99]	OR = 0.61 [0.34-1.08]	OR = 0.68 [0.40-1.16]
Engaged in agriculture activity	[21]	Parental	-	SIR = 124 [1.1-152]	-	-
Engaged in agriculture activity	[21]	Parental	-	OR = 2.44 [1.66-3.56]	OR = 1.70 [0.81-3.57]	OR = 4.21 [2.13-8.32]
Application of ≥100kg nitrogen /hectare	[21]	Parental	-	RR = 1.84 [1.22-2.76]	-	-
Farm worker	[42]	Parental	Prenatal	p = 0.35		
**Pesticide applicators**	[85]	Parental	-	OR = 1.19 [0.13-4.28]	-	-
**-**	[42]	Parental	Prenatal	p = 0.63		
**Metal worker**						
Metalworkers	[87]	Paternal	Year before conception	OR = 3.28 [1.03 - 10.52]	-	-
Metal products	[87]	Paternal	Year before conception	OR = 5.77 [1.53 - 21.77]	-	-
**Wood workers**						
Wood processors	[87]	Paternal	Year before conception	OR = 10.46 [1.20 - 91.14]	-	-
**Health related**	[36]	Paternal	Childhood	OR = 3.9 [0.4-190.7]	OR = 5.1 [0.1-405.8]	-
-	[36]	Paternal	Prenatal	OR = 1.4 [0.2-17.3]	OR = 5.2 [0.4-73.6]	-
-	[36]	Maternal	Prenatal	OR = 1.4 [0.4-5.0]	OR = 4.6 [1.1-19.1]	-
-	[87]	Maternal	Before conception	OR = 0.54 [0.26 - 1.13]	-	-
**Food producers**						
Food and beverage services industry	[87]	Paternal	Year before conception	OR = 4.36 [1.50 - 12.63]	-	-
Food products	[87]	Paternal	Year before conception	OR = 2.79 [1.34 - 5.79]	-	-
**Chemical exposures**						
**Organochlorines**						
HCB	[19]	Maternal^a^	At son’s diagnostic	OR = 4.4 [1.7–12]	-	-
p,p’- DDT	[86]	Maternal^a^	1-3 days after delivery	OR = 0.70 [0.26-1.64]	-	
o,p-DDT	[86]	Maternal^a^	1-3 days after delivery	OR = 0.77 [0.37-1.33]	-	-
p,p’- DDE	[86]	Maternal^a^	1-3days after delivery	OR = 0.19 [0.04-0.62]	-	
	[19]	Maternal^a^	At son’s diagnostic	OR = 1.3 [0.5–3.0]	-	-
Total chlordanes	[19]	Maternal^a^	At son’s diagnostic	OR = 1.9 [0.7–5.0]	-	-
Ratio p,p′-DDT/ p,p′-DDE	[86]	Maternal^a^	1-3days after delivery	OR = 3.56 [1.34-11.88]	-	
**PCB**						
Sum of PCBs	[19]	Maternal^a^	At son’s diagnostic	OR = 3.8 [1.4–10]	OR = 3.1 [0.7–14]	OR = 4.3 [1.3–14]
Estrogenic PCBs	[19]	Maternal^a^	At son’s diagnostic	OR = 2.4 [0.95–6.0]	OR = 2.3 [0.6–8.9]	OR = 2.4 [0.8–6.8]
Enzyme-inducing PCBs	[19]	Maternal^a^	At son’s diagnostic	OR = 2.6 [1.03–6.5]	OR = 1.4 [0.4–5.3]	OR = 3.3 [1.1–9.7]
Toxic equivalents (TEQ)	[19]	Maternal^a^	At son’s diagnostic	OR = 3.3 [1.3–8.4]	OR = 3.5 [0.8–15]	OR = 3.3 [1.1–9.8]
Sum of PBDE	[19]	Maternal^a^	At son’s diagnostic	OR = 2.5 [1.02–6.0]	OR = 1.8 [0.5–6.5]	OR = 2.9 [1.04–8.2]
**Endocrine-disrupting chemicals**	[44]	Maternal	Prenatal	OR = 0.97 [0.23-4.07]	OR = 0.99 [0.16-6.07]	OR = 1.13 [0.19-6.86]
-	[44]	Paternal	Prenatal	OR = 1.33 [0.65-2.70]	OR = 1.24 [0.51-3.01]	OR = 1.42 [0.55-3.67]
**Others**						
Residency urban/rural	[44]	Parental	During fetal life	OR = 1.35 [0.49-3.71]	OR = 1.54 [0.44-5.35]	OR = 1.29 [0.34-4.94]
Social class (professionals vs. manual workers)	[45]	Paternal	At birth	OR = 1.48 [0.69-3.16]	-	-

Abbreviations: HCB = Hexachlorobenzene, DDT = dichlorodiphenyltrichloroethane; DDE = Dichlorodiphenyldichloroethylene; PCB = polychlorinated biphenyl; PBDE = *Polybrominated diphenylethers*.

a Exposure assessment by biological samples, in contrast to questionnaire/registry-based exposure assessment.

### 1. Index case exposure

#### Industrial exposure

Two of the six studies on workers in plastic-related industries [22,38,46–49], found an association with a risk for TGCT [22,49]. Statistically significantly increased risk of TGCT was found for Swedish plastic industry workers exposed to polyvinyl chloride (PVC) but this association was based on analyses in a small sub-group [22]. Using a larger population, the same team observed an association between PVC and TGCT, but only in the group with the lowest level of exposure and for less than 8 years of cumulative exposure [49]. Stratification for the time between first exposure and diagnosis did not show any specific trend. The authors concluded that the association was probably due to random cluster [49]. In addition, no association with TGCT was seen for other plastic components, such as styrene, urethane or acrylate [22], as well as for chemical industry related tasks [38,48].

Five of the eight identified studies found significant associations with occupations in metal industries [31,38,50,52,53]. However, metalworking occupations and associated exposures investigated varied importantly across studies (e.g. metal trimming, metal annealer, welding) [31,38,43,48,50–53], making comparisons difficult. Occupation as a precision metal worker was found to be associated with TGCT while occupation as metal machine operators or metal making workers were not [38]. An increased risk of TGCT was found for furnace workers in a ferrosilicon plant, but no association with duration of work was reported [53]. In a nationwide study in Sweden a significantly higher risk of seminomas was reported for metal annealer and/or temperer workers, whereas no association was found for precision toolmakers, metal casters and moulders or other metal processing workers [31]. The two remaining studies showed an excess of seminomas among metalworkers [52] and of non-seminomas in a sub-group of automobile workers involved in metal-cutting tasks [50]. However the first one should be interpreted cautiously because of methodological limitations (questionnaires differed between cases and controls, no consideration of age, imprecise exposure assessment) [52]. A French case-control study reported an increased risk of TGCT for workers involved in welding and TGCT risk but this was no longer significant after adjustment for potentially confounding factors [48]. In Denmark, an increased risk of TGCT in stainless steel grinding workers was observed (based on 4 cases), but not for other metalworkers (including welders) [43]. The authors concluded that the association might be due to confounding by socio-economic status (SES). 

Seven articles investigated the association between working in the paper industry and TGCT [34,38,45,54–57] with divergent results. A Swedish registry-based cohort study reported a positive association for seminomas depending on the duration of employment [54]. This study considered only workers over 30 years of age to guarantees a minimum of cumulative exposure, thus, some TGCT cases may have been missed. Another Swedish registry-based cohort study showed a positive association for sulphate pulping workers, as well as sulphate mills workers but only for less than 10 years of exposure and latency [57]. Conversely, a Danish nationwide registry-based cohort study [56] and a Canadian cohort study did not find any association with occupation in the paper industry. Yet, the latter was based on 23 cases only [55]. The three remaining studies that investigated various occupational exposures did not find any association for paper industry workers with TGCT [34,38,45].

#### White-collar workers, professionals and higher social-economic-status (SES)

An excess risk for TGCT was reported for several white-collar or professional occupations [31,35,38]. Some authors suggested that this might be due to SES rather than occupational exposure [35,38]. Guo et al. reported an excess risk for university teachers, electrical engineers, system analysts and programmers [34] but these associations disappeared after adjustment for SES. Swerdlow et al. found an association for higher SES but not for white-collar occupations [45]. The results from Knight et al. suggested an association with SES mainly among seminoma cases [51], whereas a Danish study reported no association for SES but found an association for ethnicity [58]. 

#### Construction and related occupations

Eight studies investigated TGCT risk in construction and related occupations [22,31,35,38,45,48,59,60]. Two focused specifically on construction workers, with inconsistent results [31,35]. Electrical workers had an elevated risk in one study [38] out of four [22,38,45,60]. No association was found for painters [38,45,48]. One study on carpenters in the US reported an increased risk, but only for workers employed from 0 to 10 years, considering 15 years of latency [59]. However, comparison with the general New Jersey population might have led to an overestimation of the risk, since 97.3% of carpenters were Caucasian. In addition, no association was found for wood workers [45], lumber-jacks [38] and workers exposed to wood dust. 

#### Firemen, policeman and military workers

Five studies investigated TGCT risk in firemen [18,26,61–63] with divergent results. Excess risk was reported for fire-fighters in three studies: two cohort studies from New Zealand [18] and Florida [63] and one case-control study from California (for 1988-1995 but not for 1996-2003) [61]. Two additional studies conducted in Australia (cohort) [62] and Germany (case-control) [26] did not found any association. However, the sample sizes in these two studies were small. All five studies were registry-based and did not consider potential additional exposures. 

Four studies investigated the risk in policemen [31,33,64,65] with divergent results. Davis et al. found a TGCT cluster in Ontario and suggested this was due to electromagnetic field exposure resulting from the use of radar devices [64]. Finkelstein et al. also found a positive association, but used a 90% interval confidence[65]. A Swedish population-based study found a significant association with seminomas in policemen compared to the general population [41]. This association disappeared when compared with other occupations in the same sector (services and military work) or when policemen potentially exposed for longer periods were considered [31]. A fourth study failed to show any association [33]. 

Ten studies investigated TGCT risk in military and related occupations [20,31,37,41,45,66–70] with inconsistent results. Of the five studies investigating general military workers [20,31,45,66,69], only one reported a weak association with seminomas [31]. Five studies reported an elevated risk of TGCT for air force personnel [20,66,67,69,70] while four others did not [37,41,68] or suggested a protective effect [71]. Similar inconsistent associations were observed in studies on navy personnel [20,37,41]. Studies on American Marine Corps personnel did not report an association with TGCT [20,37,41,69]. Overall, risk excess in these studies was observed for personnel involved in specific tasks (electric, mechanic or maintenance) [20,66,69]. Non-seminoma tumours were found associated with having been in service in Vietnam [37], but not with Agent Orange exposure during the Vietnam War [41]. 

#### Farmers, agricultural workers and occupational exposures to pesticides

Overall, 15 studies investigated TGCT risk among agricultural workers, pesticides applicators or in occupations associated with pesticides exposures [31,34,35,38,42,44,45,48,51,72–77]. On the six studies assessing occupational exposures to pesticides in general, no significant associations were reported [34,35,42,44,45,51], except for one study showing a protective effect for seminoma tumours [51]. However, this study was based on self-reported exposures, small subgroups, and numerous statistical tests were performed without correction for multiple testing. On the six studies investigating agricultural occupations [31,34,35,38,45,76], only one found an increased risk, but mainly due to a small subgroup of fish farmers [76]. On the four registry-based studies focusing on licensed pesticide applicators, an increased TGCT risk was reported for a Floridian [74] and a UK study [75], but not for a Swedish study [73] or for the US Agricultural Health Study [72]. These studies may be affected by misclassification bias since unregistered agricultural workers, under the supervision of licensed farmers, may have applied the pesticides. A Norwegian study identified a slight excess risk for workers in a fertilizer production plant, but no association with exposure to a particular product [77]. Since the plant was located in a rural area, it was suggested that this was the potential impact of life-long environmental exposure to other factors, however not investigated by this study. No associations were reported for workers in the pesticide industry [48]. 

#### Magnetic and electric field exposure

Five studies investigated magnetic and electric field exposures [24,30,32,35,48]. Among these, two Swedish population-based studies reported a positive association between magnetic fields and TGCT risk (1971-1984 [30] and 1985-1987 [32]) using the same job-exposure matrix (JEM). The first reported a higher risk for the medium level of exposure and younger workers [30], the second found a dose-response effect for non-seminomas and workers under 40 years of age, but not for the others [32]. A US case-control study found a positive association for self-reported occupational exposure to microwaves and other radiowaves, but this association disappeared when the exposure was estimated independently using job titles [35]. No association was reported for radar equipment use [24,35,48] nor for working near visual display units or ‘complex electronic environment’ [24]. Radar exposures were also investigated in studies on policemen (see above), but no clear association was reported.

#### Environmental exposures to organochlorines and pesticides

Eight studies investigated environmental exposure to organochlorines using blood samples [19,27–29,42,44,78,79] or questionnaires [42,44]. The study by McGlynn et al. suggested that p,p’-DDE, oxychlordane, cis-nonachlor, trans-nonachlor and total chlordane serum levels are associated with TGCT risk, especially with seminomas [28]. Other studies did not report any association with trans-nonachlor [78,79], total chlordanes [19], and p,p’-DDE serum levels [19,42,78,79]. Two polymorphisms of the CYP1A1 gene (rs7495708 and rs1456432) were suspected to be associated with an increase of TGCT risk in men having elevated total chlordane serum levels [27]. Overall, no association was observed with serum levels of hexachlorohexane [28,78], hexachlorobenzene (HCB) [19,78], pp’DDT, oxychlordane [28,78,79], op’DDT [78,79] and mirex [28,79]. McGlynn et al. reported a protective effect for PCBs serum levels, either individually or grouped [29], while others studies showed mixed effects (increased or decreased risk) [79], or no association [19]. However, some authors suggest there is uncertainty on the conclusions to be drawn from observed association between organochlorine levels measured in adulthood and past exposures occurred during early (or prenatal) life since important physiological variations occur over life, especially at puberty [78]. Moreover, exposure may have occurred after the in utero or infancy period and genetic polymorphisms in metabolism may also have an impact on the serum concentrations [27].

One study suggested an increased risk for self-reported domestic insecticides use, but blood analyses failed to confirm this association [42]. Using a JEM, an Italian study reported an excess TGCT risk associated with hobbies involving exposure to endocrine disrupting chemicals during adolescence, but not for occupations involving the same exposure [44].

It has been suggested that living in rural areas could be a surrogate for environmental exposure to pesticides [21–23,44,48,80]. One study found an increased risk of TGCT among men who reported living in a rural area (defined as living <1 km from a farm) during adolescence [44], while others showed a protective effect [80] or no association [21,22]. Inconsistent associations with TGCT risk have also been reported for rural area residency during adulthood [22,23,44,48]. Additionally, a Swedish registry-based cohort suggested a non-significant increased risk for men living in an area contaminated by PCBs and metals [81]. 

#### Risk associated with miscellaneous occupations and exposures

Leather workers exposed to dimethylformamide (DMF) have been suspected to be at risk for TGCT based on the report of a small cluster in a New York plant [82]. Association was confirmed for non-seminomas in a second study [51], but not in two larger studies assessing various occupational exposures [34,45]. Occupations related to food processing were reported to be associated with an increased risk for TGCT in two studies investigating a wide range of occupations [38,51]. The authors suggested a role of cleaning agents, disinfectant and insecticides. Exposure to polycyclic aromatic hydrocarbons and hydrocarbons such as diesel and gasoline was not reported to be associated with a risk for TGCT [33–35,38]. Likewise exposures to radioactive material and nuclear activity were also not reported to be associated with risk for TGCT [35,48,69,83]. A US case-control study reported that worker exposed to ‘extreme temperatures’ (lower than 60°F (15°C) or higher than 80°F (27°C)) have an increased risk of TGCT, however exposure was self-reported and the response rate of cases was low (<40%) [84].

### 2. Parental exposure

Among the 9 studies investigating occupational and environmental parental exposure, agriculture-related parental exposure has been the most studied (5 studies). No excess risk for TGCT was reported among the sons of farmers or pesticide applicators [36,42,80,85], except in one study in Norway that reported an increased risk for sons of agricultural workers using nitrate fertiliser, especially with a high nitrate/phosphate ratio [21]. In this study, the adjusted risk was higher for exposed men born between April and June or October and December. Since the high nitrate/phosphate ratio is related to intensive farming, association with TGCT might be linked to specific related exposure patterns, including pesticides use, type of farming or farming practices. In a Danish study, Moller et al. suggested an increased risk associated with childhood residence in a high-nitrate area, but only for those who did not grow up on a farm [80]. Moreover, residence in a farm during childhood was associated with a protective effect. The authors concluded that nitrates are unlikely to be responsible for the observed TGCT risk excess. Another study on pesticide applicators’ children in Sweden did not report an association, however only two cases of TGCT were reported and the follow up was short (the median age at end of the follow-up was 20 to 24 years) [85]. In addition, about 25% of the sons were already born when their fathers obtained their pesticide applicator’s license. 

A positive association was found for hexachlorobenzene, PCBs, PBDE and chlordanes in maternal serum and the risk of TGCC among the women’s sons [19]. However, maternal blood samples were collected at time of sons’ TGCT diagnosis and selection bias may have occurred since cases were chosen by their physicians. In a small US nested case-control study (15 TGCT cases and 45 controls), in which the maternal blood samples have been collected during pregnancy and a few days after delivery, no association was reported between TGCT risk in sons and the mother’s DDT or DDE serum levels [86]. The authors suggested that, compared with controls, the mothers of cases may be slower to eliminate DDT. 

Other self-reported occupations in parents were reported to be associated with TGCT (e.g. healthcare professions, wood and metal-workers, working in food production) but these studies performed numerous statistical tests, had small subgroups and were exposed to possible selection bias [36,87]. Overall, in the few studies focussing on TGCT in the offspring, the methods used to assess parental exposure had limitations. 

## Discussion

To our knowledge, this is the most comprehensive review of studies published in the past two decades on environmental and occupational exposures possibly associated with TGCT, for prenatal childhood and adulthood periods. In contrast to previous reviews on TGCT [14–16,29], we considered that current evidence is inconsistent and does not allow to conclude on existence of any clear association between TGCT and adulthood occupational or environmental exposure. This is in line with current hypotheses suggesting that TGCT may originate from in utero or early life exposure [4,9]. However, very few studies investigated the impact of parental occupational or environmental exposure, and results were inconsistent. 

For the first time, this review include systematic quality appraisal for all studies. Studies reporting a positive association with TGCT had significantly lower quality scores than studies showing no association. Also, some of the inconsistent results may be explained by methodological limitations or study design, as developed below. 

### Inconsistent associations

The low incidence of TGCT constitutes a barrier to conducting adequately powered epidemiological studies. None of the publications provided information on the minimum detectable risk based on the study population size. Lack of power could partly explain the inconsistent results reported. Furthermore, some positive associations may be due to chance, when multiple testing is performed without correction (e.g. more than 300 tests were performed by Pollan et al.) [31]. Some very high Odds Ratios have been identified (e.g. OR = 14 [IC95% 2.8-75] [19]). These results might be related to outliers or interaction problems and should be interpreted with caution. Also, several publications derived from investigations within cancer registries. Since incidence excess in cancer registry leads generally to investigations, publication bias is likely because TGCT incidence would not be explored systematically in registries where incidence is normal. 

### Potential confounders

Large variations in TGCT risk have been reported for different ethnicities [89], with the highest risk being in Caucasian men. This factor was rarely assessed in the identified studies and Caucasian men could be overrepresented in some occupations such as policemen, firemen, military employees, farmers or jobs related to higher SES and might explain some associations observed. SES was generally estimated using indirect means such as income or education level and shows inconstant association with TGCT [35,45,88]. A large Danish population-based study in men over 30 years old reported no significant association for income, education level or ‘Charlson Comorbidity Index’, but only for ethnic origin [58]. Otherwise, cryptorchidism is an established risk factor for TGCT and several studies adjusted for this. However, according to the TDS hypothesis [9], cryptorchidism and TGCT may have a common underlying early developmental cause. In this case, the two factors are collinear and will have no impact on their mutual risk. Adjustment may therefore have weakened any potential association. Furthermore, several polymorphisms have been recently associated with the risk of TGCT [10,27], but rarely considered in the published studies.

### Exposure assessment

Heterogeneity of occupations and definitions of exposures may further explain inconsistencies across studies, and did not allow us to perform a pooled analysis. Moreover, while for some occupations the job title can be used as surrogate exposure variable (e.g., welders and welding fumes), the reliability of exposure assessment is limited for others (e.g., farmers and pesticides exposure). Misclassification of pesticide exposures could have resulted in weaker association since some farmers should not have been classified as exposed, whereas other occupations associated with pesticides exposure (e.g., carpenters, sawmill workers) in the general population were classified as not being exposed [90]. In addition, very few studies assessed the association with domestic exposure to pesticides (i.e. gardening, indoor use of insecticide spray) and those that did were mainly based on self-reported exposure. The whole population is exposed to ubiquitous pollutant, at least a minimum, making it difficult to identify control groups to assess related adverse effects in absence of precise exposure assessment. 

Self-reported exposures can provide detailed information, but potential recall bias may lead to over-estimation in comparison to JEM or independent assessment [91]. The evolution of industrial practices and occupational exposure could further explain inconsistency between previous studies and more recent ones.

### Plausibility of adult exposure for the index subject

TGCT occurs mainly in young adults, a population with lower cumulative occupational exposure and shorter time-lag between occupational exposure and cancer diagnosis. Thus, occupational exposure of the index subject may not be relevant. While uncertainties remain concerning the exact window of exposure associated with occurrence of TGCT, events during the ‘testicle programming period’ has been proposed to originate in cryptochidism and hypospadias in rats [92]. This is an interesting concept since these diseases are thought to have common underlying causes with TGCT in men. Extrapolating to humans, this period would correspond to the 8^th^ to 14^th^ week of pregnancy, but differences in the reproductive mechanisms of rodents and human may limit extrapolations [93,94]. 

In contrast, environmental or occupational exposure during adulthood might be associated with spermatocytic seminomas that occurring mainly after 50 years: the cumulative exposure is higher, particularly for occupational exposure, the latency periods are long enough, and the related precursor cells appeared only after puberty and during adulthood [2]. The spermatocytic seminomas subtype accounts for up to 4% of all TGCT [2] but this proportion can increase when the age limit of the sample at inclusion is higher. Since spermatocytic seminomas are generally grouped with classic seminomas, it is possible that this explains some associations between adult exposure and TGCT that are reported. To avoid potential bias, spermatocytic seminomas should be considered separately from other TGCT. 

### Limit of the review

The NOS quality assessment scale has been criticized for potential inter-operator variability [95,96]. In response to these limitations, independent evaluation by two investigators combined with arbitrage has been performed to increase reliability of scoring.

While the majority of publications in our review were identified through PubMed, additional studies were identified through the reference lists of relevant articles and recent reviews. The latter mainly focused on multiple cancer sites where TGCT was not the primary cancer of interest, and thus, not apparent in keywords of abstracts. However, we can be confident that the combination of these two methods has enabled us to identify all the pertinent studies on environmental or occupational risk factors for TGCT and the vast majority of studies exploring a wider range of cancers including TGCT.

### Recommendations

The study limitations discussed above makes it difficult to detect or to interpret associations. Future studies on TGCT should consider intra-uterine and parental exposure, since it is plausible that exposure during early development leads to TGCT and this has been insufficiently explored. A combined effect of prenatal and postnatal, adolescent or adulthood exposure has also been suggested [16], but has not been explored yet. Moreover, we recommend that domestic, environmental and occupational exposure should be assessed in future studies to minimise misclassification bias, as well as genetic and molecular biology techniques for evaluating gene–environment interactions. Quantitative exposure assessment should be improved when considering ubiquitous pollutants (e.g.: use of biomarkers or geographic information systems). Finally, to overcome problems linked with the lack of statistical power, we recommend the use of more standardized approaches in future studies to allow meta-analyses or pooled studies. This could be achieved by the creation of consortia that can give guidance on the design and analyses of next generation studies. 

A national case-control is currently conducted in France (TESTIS project) to explore the impact of life-time pesticide exposure on TGCT risk using combined methods including job exposure assessment by experts, geographical information system technology and polymorphism analyses.

## Conclusions

Despite of the numerous factors investigated in many studies, the reasons for the rapid increase of TGCT incidence remain unclear. Occupational exposures during adulthood are unlikely to be involved in TGCT aetiology because of the young age of patients. The lack of convincing association with adulthood exposure is in line with the current hypothesis of prenatal and/or early-life origin of TGCT. The limitations of the studies may partly explain the inconsistencies observed. Unfortunately, we were unable to perform a quantitative meta-analysis because of the heterogeneity in design, exposure assessment and population characteristics for the studies we identified. However, many risk factors investigated in current studies remain of interest, in particular pesticides, and recent studies highlight the potential role of gene-environment interactions. Further large studies are needed, and future research should focus on prenatal or early life exposure, as well as combined effect of prenatal and adolescent or adulthood exposure.

## Supporting Information

Table S1 **Occupational and environmental exposure related testicular germ cell cancer (publication from 1990 to 2012).**
(DOCX)

Checklist S1 **Completed PRISMA Checklist.**
(DOC)
