# Supplementary material for: Occupational and Environmental Exposures Associated with Testicular Germ Cell Tumours: Systematic Review of Prenatal and Life-Long Exposures
Source: PLoS One. 2013 Oct 14;8(10):e77130. doi: 10.1371/journal.pone.0077130 (PMC3796551; doi:10.1371/journal.pone.0077130)
Supplement: Table S1 — Occupational and environmental exposure related testicular germ cell cancer (publication from 1990 to 2012). (DOCX) [file pone.0077130.s001.docx]

**Table S1. Occupational and environmental exposure related testicular germ cell cancer (publication from 1990 to 2012).**

| **Exposures** | **Ref.** | **All testicular cancer** | **Seminoma** | **Non-seminoma** |
| --- | --- | --- | --- | --- |
| **Exposures to occupations and tasks** |  |  |  |  |
| **Plastic industry** |  |  |  |  |
| Plastics workers | [22] | OR = 3.3 [1.4-7.7] | - | - |
| Worker exposed to plastics | [46] | OR = 0.9 [0.6-1.2] | - | - |
| Manufacture of plastics | [48] | OR = 2.87 [0.84–9.77] | - | - |
| Rubber, plastic products industry | [38] | RR = 0.6 [0.1-5.7] | - | - |
| **Vinyl monochloride (VMC) or polyvinyl chloride (PVC)** | | |  |  |
| VMC | [47] | 1 Obs / 0.75 Exp [0.03-7.43] | - | - |
| PVC | [22] | OR = 6.6 [1.4-32] | OR = 5.6 [1.1-196] | - |
| PVC | [46] | OR = 0.4 [0.1-1.0] | - | - |
| PVC (1 year latency) | [49] | OR = 1.35 [1.06-1.71] |  |  |
| PVC (10 year latency) | [49] | OR = 1.45 [1.06-1.98] | - | - |
| **Metal industry** |  |  |  |  |
| Metalworkers | [52] | OR = 1.2 [0.78-1.84] | OR = 2.04 [1.13-3.68] | OR = 0.8 [0.47-1.34] |
| Metal products industry | [51] | - | OR = 0.49 [0.22-1.09] | - |
| Metal products industry | [38] | RR = 2.0 [1.0-3.8] | - | - |
| **By specific tasks** |  |  |  |  |
| Welding | [48] | OR = 2.84 [1.51-5.35] |  |  |
| Welding^a^ | [48] | OR = 1.49 [0.53-4.15]^a^ | - | - |
| Welding | [22] | OR = 0.9 [0.5-1.6] | - | - |
| Welding | [43] | SIR = 0.80 [0.47-1.26] | - | - |
| Foundry work | [48] | OR = 0.90 [0.25–3.22] | - | - |
| High dermal exposure with oil-based metal working fluids | [50] | OR = 1.87 [1.05-3.34] | OR = 1.28 [0.60-2.73] | OR = 4.72 [1.48-15.09] |
| Metal trimming | [48] | OR = 1.49 [0.53–4.15] | - | - |
| Metal annealer, temperer | [31] | - | RR = 5.85 [1.88-18.20] | - |
| Stainless steel grinders | [43] | SIR = 2.41 [0.97-4.97] | - | - |
| Furnace workers in a ferrosilicon plant | [53] | SIR = 2.3 [1.05-4.37] |  |  |
| Non furnace workers in a ferrosilicon plant | [53] | SIR = 0.72 [0.2-1.85] |  |  |
| **Paper industry** |  |  |  |  |
| Pulp and paper maintenance workers | [54] | SIR = 4.8 [1.3-12] | SIR = 6.6 [1.8-17] | - |
| Paper and printing workers | [45] | OR = 2.05 [0.84-5.02] | - | - |
| Paper products industry | [38] | RR = 0.8 [0.3-2.3] | - | - |
| Printing, publishing industry | [38] | RR = 1.0 [0.4-2.5] | - | - |
| Pulp or paper dust | [34] | OR = 2.19 [0.06–12.2] | - | - |
| Paper mill workers employees | [56] | SIR = 1.04 [0.70-1.49] | - | - |
| Pulp and paper mill workers1 | [55] | SIR = 0.96 [0.66-1.36] | - | - |
| Sulphate workers (<10y) | [57] | SIR = 1.87 [1.00-3.20] | - | - |
| Sulphate workers ( ≥10 y) | [57] | SIR = 0.84 [0.31-1.83] | - | - |
| Sulphite workers (<10y) | [57] | SIR = 1.12 [0.36-2.60] | - | - |
| **White collar** |  |  |  |  |
| Professionals (administrator, teacher, physician, veterinarian) | [35] | OR = 1.0 [0.7-1.6] | OR = 2.8 [1.4-5.4] | OR = 0.7 [0.5-1.2] |
| Administrators, manager | [38] | RR = 1.5 [1.1-2.2] | - | - |
| Administrators | [45] | OR = 1.33 [0.74-2.42] |  |  |
| Administrative and managerial | [31] | - | RR = 1.20 [0.83-1.73] | RR = 1.75 [1.09-2.82] |
| Professional and technical work | [31] | - | RR = 1.06 [0.89-1.26] | RR = 1.15 [0.91-1.46] |
| Engineer, architect, surveyor | [38] | RR = 0.8 [0.4-1.6] |  |  |
| Engineering workers | [45] | OR = 0.73 [0.52-1.03] |  |  |
| Amusement and recreation worker | [51] | - | OR = 2.15 [1.17-3.95] | - |
| Business services | [51] | - | OR = 1.66 [1.04-2.63] |  |
| Health related | [35] | OR = 1.4 [0.7-2.6] | OR = 1.9 [0.7-5.0] | OR = 1.2 [0.6-2.5] |
| Physician & other health-related jobs | [38] | RR = 5.5 [1.1-26.3] | - | - |
| School teacher | [31] | - | RR = 2.30 [1.19-4.43] | RR = 1.35 [0.43-4.20] |
| University, high education teacher | [31] | - | RR = 1.83 [0.91-3.69] | - |
| Journalist, editor | [31] | - | RR = 2.57 [1.22-5.42] | - |
| Government legislative administrator | [31] | - | RR = 1.96 [1.05-3.66] | - |
| Bookkeeping and clerical work | [31] | - | RR = 1.28 [0.95-1.72] | RR = 1.65 [1.14-2.38] |
| Professional vs manual worker | [45] | OR = 1.99 [1.14-3.47] |  |  |
| **Construction workers** |  |  |  |  |
| **Wood workers - carpenters** |  |  |  |  |
| Carpenter | [59] | SIR = 1.29 [0.78-2.01] | - | - |
| Carpenter (15y lag from entry date in the union) | [59] | SIR = 2.48 [1.29-4.32] | - | - |
| Carpenter | [38] | RR = 1.2 [0.7-2.0] | - | - |
| Construction | [35] | OR = 0.8 [0.6-1.2] | OR = 0.4 [0.2-0.9] | OR = 1.0 [0.7-1.5] |
| Construction carpenters | [34] | OR = 0.4 [0.1–1.1] | - | - |
| Woodworkers | [45] | OR = 0.74 [0.35-1.53] | - | - |
| Lumber, wood products | [38] | RR = 0.8 [0.5-1.4] | - | - |
| Wood dust | [34] | OR = 0.81 [0.26–1.89] | - | - |
| **Electrical workers** |  |  |  |  |
| Electrical workers | [45] | OR = 0.74 [0.40-1.37] | - | - |
| Electrician occupation | [38] | RR = 2.8 [1.2-6.4] | - | - |
| Electrical workers | [60] | SIR = 0.83 [0.59-1.12] | - | - |
| Electrician | [22] | OR = 1.0 [0.4-2.6] | - | - |
| Utilities in electrical power industry | [51] | - | - | OR = 3.15 [1.15-8.61] |
| **Painters** |  |  |  |  |
| Painter occupation | [38] | RR = 1.3 [0.6-3.2] | - | - |
| Industrial paints occupation | [48] | OR = 0.63 [0.18–2.25] | - | - |
| Painters and decorators | [45] | OR = 0.83 [0.35-1.97] | - | - |
| **Firemen** |  |  |  |  |
| Between 1977 and 1996 | [18] | SIR = 1.55 [0.8-2.8] | - | - |
| Between 1990 and 1996 | [18] | SIR = 2.97 [1.3-5.9] | - | - |
| Between 1988 and 2003 | [61] | OR = 1.54 [1.18-2.02] | - | - |
| Between 1988 and 1995 | [61] | OR = 1.92 [1.32-2.80] | - | - |
| Between 1996 and 2003 | [61] | OR = 1.29 [0.87-1.92] | - | - |
| Fire brigade members | [62] | SIR = 1.15 [0.13-4.17] | - | - |
| Ever worked as firefighter | [26] | OR = 4.5 [0.7-31.9] | - | - |
| Ever worked as firefighter | [63] | SIR = 1.6 [1.2-2.09] | - | - |
| **Policemen** |  |  |  |  |
|  | [64] | SIR = 6.9 (p<0.001) | - | - |
|  | [65] | OR = 1.33 [90%CI 1.0-1.75] | - | - |
|  | [31] | - | RR = 1.85 [1.05-3.28] | - |
|  | [33] | SIR = 1.10 [0.23-3.22] | - | - |
| **Military Personnel** |  |  |  |  |
| Service in royal navy | [69] | OR = 1.08 [0.51-2.28] | - | - |
| Services and military work | [31] | - | RR = 1.42 [1.08-1.86] | RR = 0.98 [0.63-1.54] |
| Armed forces | [45] | OR = 0.84 [0.53-1.33] | - | - |
| Vietnam service | [37] | OR = 2.5 [1.1-5.7] | OR = 1.8 [0.6-5.1] | OR = 2.4 [1.1-5.4] |
| **Air force** |  |  |  |  |
| Air force versus Army | [41] | OR = 1.48 [0.62-3.48] | - | - |
| Air force versus Army | [20] | OR = 1.28 [1.02-1.62] | - | - |
| Royal Air Force | [66] | OR = 3.27 [2.43-4.31] | - | - |
| Air force (Vietnam veterans) | [37] | OR = 1.8 [0.1-117.8] | - | - |
| Air force (total flight hours ≥1) | [70] | OR = 1.74 [1.04–2.92] / but no association when stratified by time |  |  |
| Aviator vs non flying officer | [67] | SIR = 1.84 [99%CI 1.19-2.86] |  |  |
| Aviator vs general population | [67] | SIR = 1.04 [99%CI 0.72-1.44] |  |  |
| Aircrew (air force & civil) | [68] | SIR = 2.51 [0.90-4.92] |  |  |
| Air force | [71] | SIR = 0.68 [0.61-0.75] |  |  |
| **Marine - navy** |  |  |  |  |
| Navy vs Army | [41] | OR = 2.60 [1.08-6.24] | - | - |
| Navy vs Army | [20] | OR = 1.29 [1.03-1.62] | - | - |
| Navy (Vietnam veterans) | [37] | OR = 3.4 [0.6- 23.8] | - | - |
| Marine | [41] | OR = 0.46 [0.19-1.07] | - | - |
| Marines versus Army | [20] | OR = 1.00 [0.71-1.41] | - | - |
| Marines in Vietnam veterans | [37] | OR = 0.7 [0.1-7.9] | - | - |
| Royal marines | [69] | OR = 0.79 [0.89-1.64] | - | - |
| Coast guard vs Army | [20] | OR = 1.31 [0.74-2.32] | - | - |
| **Others** |  |  |  |  |
| Balkans mission | [97] | SIR = 1.9 [0.8-3.7] | - | - |
| Fleet air arm vs others branches | [69] | OR = 1.90 [1.04-3.48] | - | - |
| Engineer air vs other specialties | [69] | OR = 2.32 [1.20-4.48] | - | - |
| Engineer air handler vs others | [69] | OR = 7.31 [1.81-29.53] | - | - |
| >3 days of agent orange spaying within 2km | [41] | OR = 1.39 [0.50-3.80] | - | - |
| >90 days of agent orange spaying within 8km | [41] | OR = 0.99 [0.54-1.84] | - | - |
| **Agricultural workers and pesticides users** | | |  |  |
| Agriculture/ forestry/ fishing | [35] | OR = 0.9 [0.6-1.4] | OR = 0.4 [0.2-0.9] | OR = 1.1 [0.7-1.8] |
| Agriculture/ forestry/ fishing | [31] | - | RR = 1.04 [0.78-1.38] | RR = 0.84 [0.55-1.28] |
| Agriculture/ forestry/ fishing | [45] | OR = 1.09 [0.65-1.83] | - | - |
| Agricultural workers | [31] | - | - | RR = 1.17 [0.52-2.64] |
| Agricultural workers | [34] | OR = 1.0 [0.4–2.0] | - | - |
| Farm manager | [38] | RR = 1.9 [0.6-5.4] | - | - |
| Farm worker and gardener | [38] | RR = 0.6 [0.3-1.3] | - | - |
| Farmers | [76] | SIR = 0.65 [0.26-1.33] | - | - |
| Other agriculture (fish farmers) | [76] | SIR = 3.77 [1.03-9.67] | - | - |
| Pesticides industry | [48] | OR = 0.86 [0.28–2.66] | - | - |
| Licensed pesticide applicators | [73] | SIR = 1.09 [0.68-1.67] | - | - |
| Licensed pesticide applicators | [75] | SIR = 1.26 [1.04-1.53] | - | - |
| Licensed pesticide applicators | [74] | SIR = 2.48 [1.57-3.72] | - | - |
| Commercial pesticide applicators | [72] | SIR = 1.24 [0.33-3.17] | - | - |
| Pesticides industry | [48] | OR = 0.86 [0.28–2.66] | - | - |
| **Other occupational exposures** | |  |  |  |
| Chemicals industry | [38] | RR = 1.3 [0.5-3.6] | - | - |
| Chemicals industry | [48] | OR = 1.57 [ 0.74–3.33] | - | - |
| Nitrate fertilizer workers | [77] | SIR = 226 [91-465] | - | - |
| Food and beverage processors | [51] | - | - | OR = 3.20 [1.39-7.35] |
| Food products manufacture industry | [38] | RR = 2.2 [1.0-4.9] | - | - |
| Leather products industry | [51] | - | - | OR = 4.60 [0.75-28.28] |
| Leather dyeing and tanning | [51] | - | - | OR = 4.21 [1.25-14.13] |
| Leather workers | [82] | OR = 7.2 [1.89-27.72] | - | - |
| Leather workers | [45] | OR = 1.05 [0.33-3.42] | - | - |
| Leather dust | [34] | OR = 6.77 [0.17–37.7] | - | - |
| Deck officer | [99] | SIR = 0.0[0.0-0.7] |  |  |
| Radiologic technologists | [83] | SIR = 1.32[0.76-2.13] |  |  |
| **Exposures to radiations** |  |  |  |  |
| **Magnetic & Electric fields** |  |  |  |  |
| **General** |  |  |  |  |
| Working near radiofrequency emitters | [24] | OR = 0.9 [0.60–1.24] | No difference observed | |
| Working near electrical machines | [24] | OR = 1.0 [0.72–1.33] | No difference observed | |
| Working near high-voltage lines | [24] | OR = 0.7 [0.38–1.18] | No difference observed | |
| Working in front of a visual display unit or in complex electrical environments | [24] | OR = 0.9 [0.67–1.21] | No difference observed | |
| High-voltage lines (<100 m) | [25] | OR = 1.5 [0.66-3.43] | - | - |
| High-voltage lines (>26 days) | [25] | OR = 1.8 [0.91-3.38] | - | - |
| Working near radar units | [24] | OR = 1.0 [0.60–1.75] | No difference observed | |
| Radar equipment | [35] | OR = 1.1 [0.7-1.9] | OR = 1.3 [0.6-2.8] | OR = 1.1 [0.6-1.9] |
| Occupation related to radar | [48] | OR = 0.84 [0.38–1.87] | - | - |
| Microwaves or radio waves | [35] | OR = 0.8 [0.3-2.0] | - | - |
| **Magnetic fields (using JEM)** |  |  |  |  |
| Medium level  High level | [30] | RR = 1.6 [1.2-2.1]  RR = 1.7 [1.3-2.2] | - | - |
| ≤40y in 90^th^ percentile  ≤40y in 4^th^ quartile | [32] | OR = 2.8 [1.1-6.9] (P90)  OR = 1.8 [0.9-3.4] (Q4) | OR = 1.5 [0.5-5.0]  OR = 1.1 [0.5-2.5] | OR = 4.6 [1.5-13.6]  OR = 2.9 [1.2-7.0] |
| **Ionizing radiation** |  |  |  |  |
| Radioactive material | [35] | OR = 1.2 [0.46-2.3] | OR = 1.3 [0.5-3.3] | OR = 1.2 [0.6-2.4] |
| Nuclear activity | [48] | OR = 2.13 [0.85–5.37] | - | - |
| **Exposure to chemicals** |  |  |  |  |
| **Pesticides – general** |  |  |  |  |
| Pesticides | [34] | OR = 1.28 [0.55–2.53] | - | - |
| Pesticides | [35] | OR = 1.2 [0.4-1.2] | OR = 0.1 [0.0-1.0] | OR = 1.5 [0.9-2.7] |
| Pesticides | [45] | OR = 1.04 [0.61-1.77] | - | - |
| Private pesticide use | [72] | SIR = 1.05 [0.67-1.58] | - | - |
| Pesticide or herbicide spraying | [51] | - | OR = 0.63 [0.42-0.95] | - |
| Mixing pesticides for gardening | [42] | OR = 4.80 [0.91-25.30] | - | - |
| Use of pesticide for gardening | [44] | OR = 1.22 [0.60-2.48] | OR = 0.55 [0.18-1.61] | OR = 2.23 [0.97-5.10] |
| Exposed to herbicides | [34] | OR = 1.00 [0.68–1.43] | - | - |
| Exposed to herbicides | [45] | OR = 1.14 [0.67-1.94] | - | - |
| Exposed to fungicides | [34] | OR = 1.04 [0.75–1.41] | - | - |
| Exposed to insecticides | [34] | OR = 3.05 [0.83–7.81] | - | - |
| Exposed to insecticides | [42] | OR = 3.23 [1.15-9.11] | - | - |
| **Pesticides – Serum concentrations** |  |  |  |  |
| β-hexachlorocyclohexane | [28] | OR = 0.90[0.65-1.24] | OR = 0.97 [0.63-1.49] | OR = 0.85 [0.57-1.26] |
|  | [78] | OR = 0.92 [0.51-1.64] | - | - |
| γ-hexachlorocyclohexane | [78] | OR = 1.36 [0.75-2.46] | - | - |
| Dieldrin | [78] | OR = 0.79 [0.44-1.41] | - | - |
| Hexachlorobenzene (HCB) | [78] | OR = 0.85 [0.37-1.96] | - | - |
| Heptachlor epoxide | [78] | OR = 0.67 [0.35-1.29] | - | - |
| p,p’-DDT | [78] | OR = 1.17 [0.68-2.00] | - | - |
|  | [28] | OR = 1.13 [0.71-1.82] | OR = 1.30 [0.73-2.30] | OR = 0.94 [0.50-1.77] |
|  | [79] | OR = 2.1 [0.6-7.2] | - | - |
| o,p’-DDT | [78] | OR = 1.30 [0.67-2.53] | - | - |
|  | [79] | OR = 1.4 [0.4-4.5] | - | - |
| p,p’-DDE | [78] | OR = 0.61 [0.32-1.14] | - | - |
|  | [42] | OR = 3.21 [0.77-13.30] | - | - |
|  | [28] | OR = 1.71 [1.23-2.38] | OR = 1.91 [1.22-2.99] | OR = 1.63 [1.10-2.42] |
|  | [79] | OR = 2.2 [0.7-6.5] | OR = 2.2 [0.5-8.7] | - |
| Oxychlordane | [78] | OR = 0.93 [0.50-1.73] | - | - |
|  | [28] | OR = 1.27 [0.92-1.76] | OR = 1.64 [1.04-2.60] | OR = 1.11 [0.75-1.63] |
|  | [79] | OR = 3.2 [0.6-16.8] | OR = 5.1 [0.7-36.8] | - |
| cis-nonachlor | [28] | OR = 1.56 [1.11-2.18] | OR = 1.93 [1.27-2.93] | OR = 1.32 [0.86-2.03] |
| Mirex | [28] | OR = 1.24 [0.90-1.74] | OR = 1.15 [0.75-1.77] | OR = 1.24 [0.82-1.88] |
|  | [79] | OR = 1.2 [0.4-3.0] | - | - |
| *Trans*-nonachlor | [78] | OR = 0.89 [0.49-1.61] |  |  |
|  | [28] | OR = 1.46 [1.07-2.00] | OR = 1.72 [1.11-2.67] | OR = 1.39 [0.96-2.00] |
|  | [79] | OR = 2.6 [0.7-8.9] | OR = 1.6 [0.4-6.0] | - |
| *Trans*-nonachlor + Gene CYP1A1 polymorphisms rs7495708 | [27] | OR = 1.92 [1.03–3.58] | - |  |
| *Trans*-nonachlor + Gene CYP1A1 polymorphisms rs1456432 | [27] | OR = 1.90 [1.01–3.56] | - |  |
| Total chlordanes | [28] | OR = 1.51 [1.09-2.10] | OR = 1.90 [1.20-3.00] | OR = 1.37 [0.93-2.02] |
|  | [78] | OR = 0.93 [0.51-1.68] |  |  |
|  | [79] | OR = 2.3 [0.6-7.2] | OR = 1.6 [0.4-6.6] | - |
| Total chlordanes + gene CYP1A1 polymorphisms rs7495708 | [27] | OR = 2.21 [1.17–4.15] | - | - |
| Total chlordanes + gene CYP1A1 polymorphisms rs1456432 | [27] | OR = 2.07 [1.09–3.92] | - | - |
| Total organochlorine pesticides (HCB+p,p’-DDE) | [42] | OR = 3.34 [1.09-10.17] | - | - |
| **Chlorinated biphenyls** | |  |  |  |
| PCB 99 | [29] | OR = 0.80 [0.57-1.13] | OR = 0.80 [0.51-1.25] | OR = 0.76 [0.50-1.17] |
|  | [79] | OR = 2.2 [0.8-5.9] | OR = 4.4 [1.0-20.5] | - |
| PCB 101 | [29] | OR = 1.01 [0.74-1.38] | OR = 1.12 [0.74-1.70] | OR = 0.91 [0.62-1.33] |
| PCB-118 | [29] | OR = 0.55 [0.40-0.76] | OR = 0.72 [0.47-1.12] | OR = 0.45 [0.31-0.66] |
| PCB-118 + gene HSD17B4 polymorphisms rs384346 | [27] | OR = 0.46 [0.31–0.70] | Not shown |  |
| PCB-138 | [29] | OR = 0.46 [0.32-0.66] | OR = 0.52 [0.31-0.86] | OR = 0.42 [0.27-0.65] |
| PCB-138 | [79] | OR = 1.8 [0.6–5.1] | OR = 2.1 [0.6-7.2] | - |
| PCB-138 + Gene HSD17B4 polymorphisms rs384346 | [27] | OR = 0.46 [0.30–0.72] | Not shown |  |
| PCB 153 | [29] | OR = 0.45 [0.31-0.66] | OR = 0.52 [0.31-0.87] | OR = 0.40 [0.26-0.63] |
|  | [79] | OR = 1.2 [0.4-3.4] | OR = 1.2 [0.4-4.3] | - |
| PCB 156 | [29] | OR = 0.57 [0.40-0.81] | OR = 0.54 [0.34-0.86] | OR = 0.58 [0.37-0.91] |
| PCB 163 | [29] | OR = 0.59 [0.42-0.83] | OR = 0.58 [0.37-0.92] | OR = 0.57 [0.37-0.86] |
| PCB 167 | [79] | OR = 4.4 [1.0-19.8] | OR = 6.7 [1.1-42.9] | - |
| PCB 170 | [29] | OR = 0.56 [0.39-0.80] | OR = 0.56 [0.35-0.91] | OR = 0.55 [0.36-0.85] |
| PCB 180 | [29] | OR = 0.56 [0.38-0.82] | OR = 0.67 [0.39-1.13] | OR = 0.51 [0.32-0.81] |
| PCB 183 | [29] | OR = 0.86 [0.58-1.29] | OR = 0.77 [0.46-1.29] | OR = 0.92 [0.56-1.52] |
|  | [79] | OR = 1.3 [0.5-3.5] | OR = 2.9 [0.6-13.7] | - |
| PCB 187 | [29] | OR = 0.60 [0.42-0.86] | OR = 0.75 [0.47-1.20] | OR = 0.48 [0.31-0.75] |
| Sum of PCBs (99, 101, 118, 138, 153, 156, 163, 170, 180, 183, 187) | [29] | OR = 0.46 [0.32-0.67] | OR = 0.45 [0.27-0.76] | OR = 0.45 [0.29-0.71] |
| Sum of PCB (31 congeners) | [79] | OR = 1.3 [0.5-3.8] | OR = 1.2 [0.4-4.1] | - |
| **Solvents** |  |  |  |  |
| Formaldehyde | [34] | OR = 1.03 [0.28–2.64] | - | - |
| **Hydrocarbons** |  |  |  |  |
| Diesel | [33] | OR = 1.15[0.36-3.60] | - | - |
| Diesel exhaust | [34] | OR = 1.20 [0.67–1.98] | - | - |
| Gasoline exhaust | [33] | OR = 1.58 [0.22-11.4] | - | - |
| Gasoline engine exhaust | [34] | OR = 1.02 [0.44–2.01] | - | - |
| Gasoline | [34] | OR = 1.19 [0.44–2.59] | - | - |
| Polycyclic aromatic hydrocarbons/ combustion/ drilling of fossil fuels | [35] | OR = 1.5 [0.7-3.4] | OR = 1.0 [0.2-3.9] | OR = 1.7 [0.7-4.0] |
| Petroleum/coal refining & products industry | [38] | RR = 1.1 [0.2-7.3] | - | - |
| **Other exposures** |  |  |  |  |
| **temperature** |  |  |  |  |
| Extreme (<60 F° or >80 F°) | [84] | OR = 1.71 [1.13-2.60] | - | - |
| Low temperature (<60 F°) | [84] | OR = 1.70 [1.04-2.78] | - | - |
| High temperature (>80F °) | [84] | OR = 1.20 [0.80-1.80] | - | - |
| **Residency** |  |  |  |  |
| **Adulthood** |  |  |  |  |
| Urban vs rural | [22] | OR = 1.5 [0.9-2.4] | - | - |
| Rural vs urban | [44] | OR = 1.27 [0.32-5.05] | OR = 1.40 [0.27-7.09] | OR = 1.58 [0.26-9.75] |
| Rural vs urban | [48] | OR = 1.63 [1.16-2.29] | - | - |
| Rural vs urbain^a^ | [48] | OR = 1.43 [0.83–2.46]^a^ | - | - |
| very dense urbanization (≤2500 hab/km^2^) vs. rest of the Netherlands | [23] | IR = 4.4 (NS)^b^ | IR = 2.3 (NS)^b^ | IR = 1.8 (NS)^b^ |
| Very low urbanization (<500 hab/km^2^) vs. rest of the Netherlands | [23] | IR = 4.4 (NS)^b^ | IR = 2.3 (NS)^b^ | IR = 1.8 (NS)^b^ |
| Living in area exposed to metals and PCBs | [81] | SIR = 2.46 [0.99-2.42] | - | - |
| **Childhood - Adolescence** |  |  |  |  |
| Urban vs rural (childhood) | [22] | OR = 1.3 [0.8-2.0] | - | - |
| Childhood in area with ≥ 6 fungal warning a year | [98] | RR = 1.2[0.7-2.1] | - | - |
| Childhood in the country | [80] | OR = 0.79 [0.63-1.00] | OR = 0.85 [0.63-1.13] | OR = 0.71 [0.52-0.98] |
| Childhood in high-nitrate area | [80] | OR = 1.40 [1.09-1.81] | OR = 1.36 [1.00-1.86] | OR = 1.49 [1.06-2.08] |
| Rural vs urban at adolescence | [44] | OR = 5.73 [1.26-25.97] | OR = 12.14 [2.14-68.78] | OR = 1.47[0.18-11.81] |
| More than 6 month in a farm | [80] | OR = 0.72 [0.55-0.94] | OR = 0.86 [0.62-1.19] | OR = 0.57 [0.39-0.83] |

*Abbreviations: y = year; JEM = job exposure matrix; DDT = dichlorodiphenyltrichloroethane; DDE= Dichlorodiphenyldichloroethylene.*

*a: Adjusted for environmental and occupational exposures and reproductive health history.*

*b: Association was declared to be not significant but confidence interval was not shown.*

*If not specified, confidence interval (IC) is 95%.*
